# Risk factors for depressed mood amongst a community dwelling older age population in England: cross-sectional survey data from the PRO-AGE study

**DOI:** 10.1186/1471-2318-14-5

**Published:** 2014-01-23

**Authors:** Jane P Biddulph, Steve Iliffe, Kalpa Kharicha, Danielle Harari, Cameron Swift, Gerhard Gillmann, Andreas E Stuck

**Affiliations:** 1 Research Department of Epidemiology and Public Health, UCL, 1-19 Torrington Place, London WC1E 6BT, UK; 2 Research Department of Primary Care and Population Health, UCL, London, UK; 3 Department of Ageing and Health, St. Thomas’ Hospital, London, UK; 4 Clinical Age Research Unit, Kings College London, London, UK; 5 Department of Social and Preventive Medicine, University of Bern, Bern, Switzerland; 6 Department of Geriatrics, Inselspital and University of Bern Hospital, Bern, Switzerland

**Keywords:** Case finding, Depression, Older people, Ageing, Vision loss

## Abstract

**Background:**

The Quality and Outcomes Framework in the United Kingdom (UK) National Health Service previously highlighted case finding of depression amongst patients with diabetes or coronary heart disease. However, depression in older people remains under-recognized. Comprehensive data for analyses of the association of depression in older age with other health and functional measures, and demographic factors from community populations within England, are lacking.

**Methods:**

Secondary analyses of cross-sectional baseline survey data from the England arm of a randomised controlled trial of health risk appraisal for older people in Europe; PRO-AGE study. Data from 1085 community-dwelling non-disabled people aged 65 years or more from three group practices in suburban London contributed to this study. Depressed mood was ascertained from the 5-item Mental Health Inventory Screening test. Exploratory multivariable logistic regression was used to identify the strongest associations of depressed mood with a previous diagnosis of a specified physical/mental health condition, health and functional measures, and demographic factors.

**Results:**

Depressed mood occurred in 14% (155/1085) of participants. A previous diagnoses of depression (OR 3.39; P < 0.001) and poor vision as determined from a Visual Function Questionnaire (OR 2.37; P = 0.001) were amongst the strongest factors associated with depressed mood that were independent of functional impairment, other co-morbidities, and demographic factors. A subgroup analyses on those without a previous diagnosis of depression also indicated that within this group, poor vision (OR 2.51; P = 0.002) was amongst the strongest independent factors associated with depressed mood.

**Conclusions:**

Previous case-finding strategies in primary care focussed on heart disease and diabetes but health-related conditions other than coronary heart disease and diabetes are also associated with an increased risk for depression. Complex issues of multi-morbidity occur within aging populations. ‘Risk’ factors that appeared stronger than those, such as, diabetes and coronary heart disease that until recently prompted for screening in the UK due to the QOF, were identified, and independent of other morbidities associated with depressed mood. From the health and functional factors investigated, amongst the strongest factors associated with depressed mood was poor vision. Consideration to case finding for depressed mood among older people with visual impairment might be justified.

## Background

Depression in older people is mainly treated within primary care but less than half of cases are being recognized [1,2]. A significant proportion of the older population aged 65 years or more are affected by major depression with a prevalence of 9% [3]. Subthreshold (subsyndromal or ‘minor’) depression is generally, at least 2-3 times more prevalent than major depression, with approximately 8-10% of those with subthreshold depression developing major depression each year [4].

Depression or psychological distress may play an aetiological role in the development of co-morbid conditions such as coronary heart disease and stroke [5,6], and a chronic physical health problem can both cause and exacerbate depression [7]. An increased risk for depression has been observed with health problems including heart disease [8], subjective experience of pain or conditions associated with pain, such as, arthritis [8,9].

Recognition of depression is important if it is followed with effective treatment and management as there is strong evidence to suggest that treating depression in older people improves outcomes [10,11], and is potentially cost effective [11]. The question is how best to enhance recognition of depression. One answer is to screen for this in individuals at higher risk of having it. Previously, a case-finding approach to late-life depression was adopted in the United Kingdom (UK). The Quality and Outcomes Framework (QOF) was introduced in the National Health Service in the UK in 2004-2005, and from April 2006 until March 2013 included case finding for depression amongst patients on the diabetes or coronary heart disease registers using simple screening questions. This recognised the increased risk for depression amongst those with coronary heart disease or diabetes compared with the general population, and the association of co-morbid depression with poorer prognosis [12]. However, despite previous focus, it is important to recognise that health problems and factors other than coronary heart disease and diabetes might also be associated with an increased risk for depression requiring treatment. Clinical guidelines recommend alertness to possible depression, particularly in patients with a past history of depression or a chronic physical health problem associated with functional impairment [7]. Further consideration of the associations of depression with other health problems including functional impairment could enhance recognition. However, comprehensive analyses of data on depression, demographic, health and functional measures from older community populations within England are lacking.

In this study, we investigated the association of depressed mood with health conditions, and whether this was independent of functional impairment, in an older age community dwelling population in England. We used this population to identify from amongst a set of health and functional measures those associated with the strongest ‘risk’ for depressed mood within this population, and within a subgroup without a previous history of depression. To assess the robustness of the selection of ‘risk’ factors, we also investigated these identified factors within an additional older age sample.

## Methods

### Participants

The analytical samples were derived from the PRO-AGE study, which was the first large-scale randomised controlled trial of health risk appraisal for older people in Europe [13], and used a self-completion multidimensional Health Risk Appraisal instrument for elderly people adapted for use in Europe by the Geriatrics Research Unit of Bern, Switzerland, in collaboration with other institutions [14,15].

In the PRO-AGE study, subjects were recruited between 2000 and 2001 from three large GP group practices (18 general practitioners) in London participating in a multi-national randomised controlled trial investigating the effect of Health Risk Appraisal for Older persons. Eligibility criteria were patients aged 65 years and over; who were living at home without evidence of need for human assistance in performing basic activities of daily living (BADL); without cognitive impairment, or a terminal illness; who were able to speak English; and who fully completed and returned a postal Probability of Recurrent Admissions questionnaire [16,17], and a consent to participate form. The Probability of Recurrent Admissions questionnaire is a screening instrument used to identify members of older populations who are at risk for using health services heavily in the future. The general goal of the trial was to evaluate intervention through the use of the Health Risk Appraisal for Older persons (HRA-O) questionnaire, feedback reports to older people and providers, and personal patient education [13]. The details of the design of this trial and questionnaire are given elsewhere [13,15].

**Figure 1:**
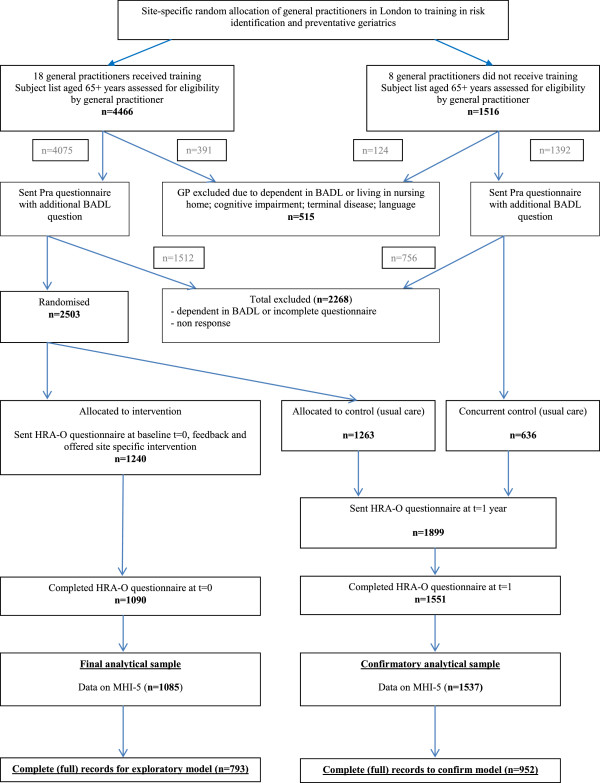
Derivation of the analytical samples from the PRO-AGE study: a randomised controlled trial of Health Risk Appraisal for older persons based in general practice*. *For further information on trial design see reference 13. HRA-O = Health Risk Appraisal for Older persons.

A flow diagram explaining the derivation of the analytical samples used in these analyses using data from the PRO-AGE study are given in Figure 1. Of an initial list of 4466 subjects aged 65 years and older who were initially assessed for eligibility, 4075 (91%) were sent a Probability of Recurrent admissions questionnaire, which also included an additional BADL question to exclude the most disabled. Of the 4075, 117 (3%) were excluded as they were dependent in BADL, 163 (4%) returned incomplete questionnaires, 1292 (32%) were non responders, leaving 2503 (61%) eligible for randomisation. Of these, 1240 (50%) were allocated to the intervention group who were sent a HRA-O questionnaire at baseline (t = 0). The remaining, 1263 subjects were allocated to the control group of usual care and were sent a HRA-O questionnaire one year from baseline (t = 1). The 1090 (88%) subjects who completed and returned this self-administered HRA-O questionnaire at baseline were included in analyses, and used data from this baseline questionnaire.

For confirmatory analyses, a second sample was identified. The sample was derived from the two control arms of this trial who were sent the HRA-O questionnaire one year after baseline (t = 1), but not at t = 0 (see Figure 1). This included data from 1263 subjects who were randomly allocated to the control group of usual care, and 636 subjects who constituted a concurrent London based comparison group (1 large group practice; 8 general practitioners) within this trial.

### Measurement of the outcome and risk factors

Data on the outcome of depressed mood, and health, function, and demographic ‘risk’ factors were selected for analyses from the HRA-O questionnaire; age at time of completion of questionnaire, sex, ethnic group, functional change, functional decrease, instrumental activities of daily living (IADL) and health that included chronic health problems, pain, self-perceived general health, vision related difficulties and hearing related difficulties screening scores.

Depressed mood was ascertained from the 5-item Mental Health Inventory Screening test (MHI-5) [18]. It asks questions about how the person felt during the past month. It has been included as one of the subscales of the RAND 36-item health survey 1.0 (distributed by RAND) and the Short Form-36 (SF-36) [19] and have equivalent summary scores of between 0-100 for the subscale [20]; 0 (poor mental health status) to 100 (good mental health status). A score of ≤ 65 was used to indicate depressed mood, and has been found to have a sensitivity of 87% and a specificity of 70% for detecting mood disorders [21]. A further five participants were excluded who had missing data on depressed mood, consequently, reducing the number of subjects contributing to analyses to 1085 subjects.

Ethnic group was self-reported according to one of ten categories. For analyses, these were grouped into ‘White UK’ and ‘Other’. Chronic health problems were determined from ‘yes’ or ‘no’ responses to the survey question ‘Has a doctor ever told you that you have…’ high blood pressure; coronary heart disease, heart attack, or heart failure; stroke; chronic bronchitis or emphysema; asthma; arthritis or rheumatism; osteoporosis; diabetes; depression; emotional or mental illness other than depression; glaucoma; irreversible/untreatable retinal disease; and cataracts. Pain was self-reported and categorised as either ‘no pain’ or ‘pain’ over the last 4 weeks. The self-perceived general health of participants was self-reported as one of four categories; excellent, good, fair or poor [22]. The categories excellent and good, were combined, as were, fair and poor for analyses. Functional change and functional decrease were each based on responses to three questions; whether for health or physical reasons, in the past 12 months, the participant either ‘changed the way’ (for functional change) or ‘decreased how often’ (for functional decrease) they walk ½ mile, climb 10 steps or get into or out of a car or bus, a positive response to any of the three questions was recorded as a functional change/functional decrease [23]. IADL [24] categories were based on whether or not there was a difficulty or need for human assistance in ≥ 1 IADL items. Vision related difficulties screen scores were ascertained from a shortened version [15,25] of the National Eye Institute Visual Function Questionnaire (VFQ) [26]. Eight items were included and scored and included items on general health and vision, difficulty with activities, and responses to vision problems [13]. Subjects were classified as having ‘VFQ-poor vision’ or ‘VFQ-not poor vision’. Poor vision included those describing their eyesight as ≥ very poor eyesight, having ≥ moderate difficulty with an activity, limited because of eyesight most or all of the time, or having given up driving mainly because of eyesight or both eyesight and other reasons. Hearing related difficulties screen results were scored and based on the Hearing Handicap Inventory for the Elderly with a score ≥ 10 indicating a hearing difficulty [27], or a response of ‘poor’, very poor’, or ‘deaf’, to an additional question of ‘How would you rate your hearing (with your hearing aid on, if applicable)?’.

### Statistical analyses

Univariable logistic regression was used to derive odds ratio (OR) estimates and 95% confidence intervals (CI) of the likelihood of depressed mood with demographic, health, functional, vision and hearing related difficulties screen variables. To assess the contribution of functional impairment on the association of an array of health conditions with depressed mood, models adjusting for functional impairment; functional decrease and difficulty or need for human assistance in ≥ 1 IADL items, were also derived. Exploratory analyses were performed whereby multivariable logistic regression using both forward selection, and backward elimination, were used to select demographic, health and functional measures independently associated with depressed mood, with and without adjustment for functional impairment within this population and within a subgroup without a previous history of depression. On multivariable analyses, it was ascertained that variable selection was the same using either forward selection or backward elimination. Univariable and multivariable analyses of data from 793 respondents with complete records were performed and repeated using all available data from 1085 respondents. Findings for complete record analyses were similar. The results present data from analyses utilising all available data. To assess the robustness of the selection of ‘risk’ factors identified from exploratory multivariable models using baseline data (t = 0), confirmatory analyses using a second sample was used to confirm the significance and independence of those ‘risk’ factors identified within a multivariable model. All analyses used STATA/SE 12.1 (StataCorp, Texas, USA) and differences were deemed significant if the *P* value was less than 0.05.

## Results

Of the 1240 subjects randomised to the group to receive the baseline questionnaire, and who had already previously returned a completed Probability of Recurrent admissions questionnaire, 150 (12%) did not return the baseline questionnaire. The responders and non-responders were evenly matched, in terms, of mean age (74.7 versus 74.8 years) and sex (45% versus 42% male).

Depressed mood occurred in 14% (155/1085) of responders with females having a 2.1-fold increased likelihood of depressed mood [OR 2.09, 95% CI 1.45-3.01, *P* < 0.001] compared with males (Table 1). Of the thirteen self-reported previously doctor diagnosed physical/mental health conditions, univariable analyses indicated that a previous diagnosis of depression [OR 4.52, 95% CI 2.98-6.87, *P* < 0.001], followed by glaucoma [OR 3.59, 95% CI 2.05-6.29, *P* < 0.001] were associated with the strongest ‘risk’ for depressed mood (Table 2). A previous diagnosis of arthritis or rheumatism (*P* = 0.04), osteoporosis (*P* = 0.001) and cataracts (*P* = 0.02) were also associated with a significantly increased risk for depressed mood with odds ratios between 1.45 and 2.34. The association of depressed mood with either diabetes [OR 1.28, 95% CI 0.70-2.33, *P* = 0.430], or coronary heart disease/heart attack/heart failure [OR 1.28, 95% CI 0.79-2.06, *P* = 0.312], failed to reach significance. Of the other health (pain; self-perceived general health; vision and hearing related difficulties screen) and functional (functional change; functional decrease; IADL) measures, all were highly significantly associated with depressed mood (P < 0001), with the ‘risk’ for depressed mood being 4-times higher amongst those with VFQ-poor vision, and having fair or poor self-perceived general health.

**Table 1:** Association between depressed mood and demographic factors: univariable logistic regression analyses, and logistic regression analyses adjusting for functional impairment (n ≤ 1085)

			**Likelihood of depressed mood**		Likelihood of depressed mood adjusted for functional impairment**	
	**% with depressed mood (no. with depressed mood/no. of subjects)**	Association with depressed mood ( *p *)*	**Odds ratio**	**95% CI**	**Odds ratio**	**95% CI**
Total	14 (155/1085)					
Age		0.125				
65-74 years	13 (77/604)		1.00	-	1.00	-
16 (78/481)		1.32	0.94 – 1.86	0.85		
Sex		<0.001				
Male	10 (47/490)		1.00	-	1.00	-
Female	18 (108/595)		2.09	1.45 – 3.01	1.70	1.14 – 2.53
Ethnic group		0.245				
White UK	14 (130/945)		1.00	-	1.00	-
Other	18 ( 21/115)		1.40	0.84 – 2.33	1.65	0.97 – 2.80

*Chi-squared with Yates’ continuity correction; **Adjusted for functional decrease and instrumental activities of daily living.

**Table 2:** Association between depressed mood, and health problems and function: univariable logistic regression analyses, and logistic regression analyses adjusting for functional impairment (n ≤ 1085)

			**Likelihood of depressed mood**		Likelihood of depressed mood adjusted for functional impairment***	
	**% with depressed mood (no. with depressed mood/ no. of subjects)**	Association with depressed mood ( *p *)*	**Odds ratio**	**95% CI**	**Odds ratio**	**95% CI**
**Self-reported previously diagnosed physical/mental health conditions**						
High blood pressure		0.612				
No	14 (76/561)		1.00	-	1.00	-
Yes	15 (76/513)		1.11	0.79 – 1.56	1.00	0.69 – 1.44
Coronary heart disease, heart attack or heart failure		0.376				
No	14 (128/933)		1.00	-	1.00	-
Yes	17 (24/142)		1.28	0.79 - 2.06	1.03	0.61 - 1.73
Stroke		0.187				
No	14 (144/1027)		1.00	-	1.00	-
Yes	22 (10/45)		1.75	0.85 – 3.62	1.12	0.50 – 2.53
Chronic bronchitis or emphysema		0.285				
No	14 (143/1028)		1.00	-	1.00	-
Yes	21 (9/43)		1.64	0.77 – 3.49	0.90	0.36 – 2.24
Asthma		1.000				
No	14 (130/919)		1.00	-	1.00	-
Yes	14 (21/149)		1.00	0.61 – 1.64	0.75	0.43 – 1.33
Arthritis or rheumatism		0.043				
No	12 (77/622)		1.00	-	1.00	-
Yes	17 (75/442)		1.45	1.02 – 2.04	1.18	0.80 – 1.74
Osteoporosis		0.001				
No	13 (127/980)		1.00	-	1.00	-
Yes	26 (23/89)		2.34	1.41 – 3.90	1.74	0.99 – 3.06
Diabetes		0.542				
No	14 (140/994)		1.00	-	1.00	-
Yes	17 (14/81)		1.27	0.70 – 2.33	0.95	0.50 – 1.82
Depression		<0.001				
No	11 (106/948)		1.00	-	1.00	-
Yes	36 (45/124)		4.52	2.98 – 6.87	4.25	2.73 – 6.62
Emotional or mental illness other than depression		0.064**				
No	14 (141/1037)		1.00	-	1.00	-
Yes	26 (8/31)		2.21	0.97 – 5.04	1.94	0.83 – 4.52
Glaucoma		<0.001				
No	13 (132/1011)		1.00	-	1.00	-
Yes	35 (21/60)		3.59	2.05 – 6.28	3.34	1.82 – 6.14
Irreversible/untreatable retinal disease		0.421**				
No	14 (146/1042)		1.00	-	1.00	-
Yes	20 (6/30)		1.53	0.62 – 3.82	1.05	0.35 – 3.16
Cataracts		0.019				
No	13 (109/848)		1.00	-	1.00	-
Yes	19 (43/223)		1.62	1.10 – 2.39	1.24	0.81 – 1.91
**Other health and functional measures**						
Pain		<0.001				
No pain	9 (56/614)		1.00	-	1.00	-
Pain	19 (76/402)		2.32	1.60 – 3.37	1.96	1.31 – 2.95
Self-perceived general health		<0.001				
Excellent or good	10 (81/839)		1.00	-	1.00	-
Fair or poor	30 (70/237)		3.92	2.73 – 5.63	2.78	1.84 – 4.21
Functional change		<0.001				
No	9 (45/504)		1.00	-	1.00	-
Yes	19 (102/541)		2.37	1.63 – 3.45	1.29	0.77 – 2.17
Functional decrease		<0.001				
No	9 (60/649)		1.00	-	-	-
Yes	22 (84/378)		2.80	1.96 – 4.02	-	-
IADL		<0.001				
Without difficulty or need for human assistance	10 (71/680)		1.00	-	-	-
With difficulty or need for human assistance	20 (75/368)		2.20	1.54 – 3.13	-	-
Vision related difficulties screen		<0.001				
VFQ-not poor vision	10 (88/843)		1.00	-	1.00	-
VFQ-poor vision	33 (58/178)		4.15	2.83 – 6.08	3.44	2.25 – 5.25
Hearing related difficulties screen		<0.001				
Not poor hearing	12 (92/788)		1.00	-	1.00	-
Poor hearing	22 (45/203)		2.15	1.45 – 3.20	1.89	1.24 – 2.88

*Chi-squared with Yates’ continuity correction; **2-sided Fisher’s exact test; ***Adjusted for functional decrease and IADL difficulties.

Functional impairment appeared to explain some of the association of health conditions with depressed mood with odds ratios for health problems reduced after adjustment. The odds ratio for depressed mood with either diabetes or coronary heart disease/heart attack/heart failure became close to 1.0. A previous history of arthritis or rheumatism, osteoporosis, and cataracts that were significant on univariable analyses were no longer significant.

Exploratory multivariable analyses identified a previous diagnosis of depression or glaucoma, VFQ-poor vision, self-perceived fair or poor general health, poor hearing, female, and being in pain as significant and independently associated with depressed mood (Table 3). This remained significant after adjustment for functional impairment, which had little impact on the ORs within the multivariable model. The strongest independent predictors were a previous diagnosis of depression, glaucoma, or VFQ-poor vision with odds ratios of 2.4 and above. Subgroup analysis of those without a self-reported previous diagnosis of depression, being female was no longer a significant independent variable. Poor hearing was significant after adjustment for functional impairment, whereas fair or poor self-perceived general health was not within this group. A previous diagnosis of glaucoma, VFQ-poor vision, and being in pain were significantly and independently associated with depressed mood amongst those without a self-reported previous diagnosis of depression as also found amongst all subjects. Within the confirmatory analyses, all variables within the multivariable models remained significant except a previous diagnosis of glaucoma.

**Table 3:** Independent factors associated with depressed mood: multivariable logistic regression analyses

	**Likelihood of depressed mood**		**Likelihood of depressed mood in a model also adjusted for functional decrease and IADL difficulties**	
	**Odds ratio**	**95% CI, *p*-value**	**Odds ratio**	**95% CI, *p*-value**
**All subjects (n = 793)**				
Depression				
No	1.00	-	1.00	-
Yes	3.39	1.93 – 5.96, *p* < 0.001	3.40	1.93 – 5.97, *p* < 0.001
Glaucoma				
No	1.00	-	1.00	-
Yes	2.39	1.06 – 5.40, *p* = 0.036	2.38	1.05 – 5.38, *p* = 0.038
Vision screen				
VFQ-not poor vision	1.00	-	1.00	-
VFQ-poor vision	2.37	1.41 – 3.99, *p* = 0.001	2.34	1.38 – 3.97, *p* = 0.002
Self-perceived general health				
Excellent or good	1.00	-	1.00	-
Fair or poor	2.17	1.31 – 3.61, *p* = 0.003	2.12	1.24 – 3.62, *p* = 0.006
Hearing screen				
Not poor hearing	1.00	-	1.00	-
Poor hearing	1.87	1.12 – 3.11, *p* = 0.016	1.87	1.12 – 3.11, *p* = 0.016
Sex				
Male	1.00	-	1.00	-
Female	1.74	1.07 – 2.83, *p* = 0.025	1.72	1.04 – 2.83, *p* = 0.033
Pain				
No pain	1.00	-	1.00	-
Pain	1.17	1.04 – 1.32, *p* = 0.009	1.17	1.04 – 1.32, *p* = 0.012
**No self-reported previous diagnosis of depression (n = 710)**				
Glaucoma				
No	1.00	-	1.00	-
Yes	2.70	1.16 – 6.31, *p* = 0.022	2.98	1.28 – 6.98, *p* = 0.012
Vision screen				
VFQ-not poor vision	1.00	-	1.00	-
VFQ-poor vision	2.51	1.39 – 4.53, *p* = 0.002	2.25	1.23 – 4.12, *p* = 0.009
Self-perceived general health				
Excellent or good	1.00	-	-	-
Fair or poor	1.98	1.12 – 3.49, *p* = 0.018	-	-
Hearing screen				
Not poor hearing	-	-	1.00	-
Poor hearing	-	-	1.78	1.02 – 3.12, *p* = 0.043
Pain				
No pain	1.00	-	1.00	-
Pain	1.21	1.06 – 1.38, *p* = 0.005	1.21	1.06 – 1.38, *p* = 0.005

VFQ-poor vision occurred in 17% (178/1021) of individuals. Of those with a previous diagnosis of glaucoma, 51% (29/57) were defined as having VFQ-poor vision. Previously within England, case finding for depression occurred amongst patients on the diabetes or coronary heart disease registers. From our data, of those with either diabetes or coronary heart disease/heart attack/heart failure (n = 211), there were 34 cases of depressed mood; 22% of all cases of depressed mood. Clinical guidelines recommend alertness to possible depression in patients with a past history of depression. Amongst those with a previous diagnosis of depression (n = 124), there were 45 cases of depressed mood; 29% of all cases of depressed mood. Extending this to also include those with VFQ-poor vision (n = 259), would increase the number of cases of depressed mood that might be identified to 78 cases (50% of all cases).

## Discussion

This study explored the association of a large array of comorbid health and functional measures with depressed mood as determined by the MHI-5. Complex issues of multi-morbidity occur within aging populations, and a range of health and functional measures appeared to identify older people at a high risk for depression. ‘Risk’ factors that appeared stronger than those, such as, diabetes and coronary heart disease that until recently prompted for screening in the UK due to the QOF, were identified.

Functional impairment explained some of the association of co-morbid health with depressed mood with odds ratios tending towards one but not all; being female, a previous doctor diagnosis of depression, and glaucoma, VFQ-poor vision, hearing difficulties, pain within the last 4 weeks, and fair or poor self-perceived general health remained significantly associated with depressed mood. Within the multivariable model, all of these factors remained significantly, and independently associated with depressed mood. Amongst the strongest ‘risk’ factors were a previous diagnosis of depression, glaucoma, and VFQ-poor vision, and amongst those without a previous diagnosis for depression, these were glaucoma and VFQ-poor vision. The association between sensory impairment and depression has been noted by other studies [28,29]. Capella-McDonnall [28], noted significant associations of self-reported single sensory loss (vision loss or hearing loss) with depression, and that depression was more frequently associated in those with vision loss compared with hearing loss, which was also indicated in our study.

Approximately, half of those with glaucoma also had VFQ-poor vision. It may be that there were aspects to the visual difficulties within subjects with glaucoma that have not been captured by the current vision screen, or may have been remedied with current treatment and might reflect self-perception. Some aspects of visual function may be more associated with depressed mood than others [30]. There are limited data from studies that investigate glaucoma and depression. However, a recent study indicated that its association did not remain significant after adjustment for self-reported general health [31]. This was not observed within our population, and glaucoma remained significant after adjustment utilising baseline data. However, the association of glaucoma with depression was not confirmed utilising data from the control arms of this trial. This aspect potentially warrants further investigation due to the low number of cases within our study, paucity of data and conflicting findings. Retinal disease failed to reach significance on univariable analyses, and may, in part, be due to low numbers as this condition was associated with the lowest lifetime prevalence. However, the questionnaire framed this as doctor diagnosed ‘untreatable/irreversible’ retinal disease, and this may have excluded some respondents with diagnosed retinal disease. However, no further specific retinal disorder questions were asked within the questionnaire.

Both poor vision and poor hearing are common chronic conditions in later life and the potential impact of these conditions on mood are of concern as the population ages. Poor social support [32], or economic well-being and social and civic participation [33] may, in part, explain some of the association between self-reported visual impairment and depression. These factors were not investigated within our model, and potentially warrant further investigation.

### Strengths and limitations of the study

This study used data from the HRA-O questionnaire, which is a comprehensive questionnaire that uses standardized and validated instruments. Rich data from a large community-dwelling non-disabled population of older adults in England were utilised, and comprehensive analyses adjusting for potential explanatory variables was undertaken. The MHI-5 screens for those with depressed mood, and although it is widely used to measure quality of life, it has received less attention within the mental health literature. However, it performs well in detecting a variety of psychiatric disorders, including mood disorders or major depression [21,34], and has been recommended for use to measure psychological distress within a European framework [35]. The findings are based on data from a limited geographical area, and participants could be considered to be the ‘healthy’ older population. Generalisability to wider areas beyond those of our London based sample and settings requires consideration.

The study uses survey data which were self-reported, and participants are those who self- completed and returned the health risk appraisal questionnaire, and is subject to bias. Responses to self-reported measures of vision, hearing, pain, and self-reported general health might be influenced by ‘mood’. Self-rating of general health reflects a global assessment of health incorporating physical, mental and social factors, albeit the strength of association may be greater for physical functioning [36]. Whilst the study can investigate the strengths of associations, it cannot investigate the temporal association of health, functional, and demographic variables with depressed mood, and therefore, the directionality of association. However, there are potentially aspects of these variables that are distinct from one another as implied by their independence within the multivariable model.

The questionnaire determines self-reported lifetime prevalence of a condition, and whilst previously diagnosed conditions investigated in this study might be considered as chronic conditions, the possibility of diminishing symptomatology from the time of the last episode or correction of a condition, such as, surgery for cataracts, cannot be excluded, and might complicate comparisons with other data investigating the effects of co-morbidity. Multiple testing and therefore the risk of a significant result arising by chance within the current analyses also needs to be considered and interpretation through consideration to the p-values advisable. However, additional confirmatory analyses of the importance of the variables within the multivariable models identified utilising baseline data was undertaken using data from the control arms of this trial.

## Conclusions

This study highlights that, in older adults, those with poor vision indicated through self-reported measures or glaucoma, were at significantly increased risk for depressed mood, independent of functional impairment, other co-morbidities, and demographic factors. Alertness to possible depression and consideration of case finding for depressed mood to older people with visual impairment may be justified, especially as this group may be less likely to have their depression recognised [37]. However, further investigation of the association of glaucoma and depression is required due to conflicting data.

If sensory impairment were causally linked directly or indirectly with depression, there is the possibility of addressing remedial factors, such as, the majority of those with cataracts causing visual impairment are not in touch with the eye services [38]. Perceptions of visual health may also play an important role in identifying depressed mood amongst the older population.

### Ethics committee

Approval was obtained from Brent Medical Ethics Committee (BEC 745) and King’s College Hospital Research Ethics Committee (01-010).

## Competing interests

All authors declare that they have no competing interests.

## Authors’ contributions

AS, KK, DH, CS, GG, and SI were members of the PRO-AGE project group. KK, DH, SI and CS implemented the London (UK) trial. JB conceptualised, undertook the analyses, drafted the original version and subsequent drafts of the manuscript on which authors commented. JB and SI are the guarantors for the paper. All authors read and approved the final manuscript.

## Pre-publication history

The pre-publication history for this paper can be accessed here:

http://www.biomedcentral.com/1471-2318/14/5/prepub
